# Adherence and Satisfaction With Intensive Physiotherapy Treatment During Ongoing Chemotherapy Sessions in Patients With Chest Wall Ewing Sarcoma

**DOI:** 10.7759/cureus.52289

**Published:** 2024-01-15

**Authors:** Vaishnavi M Thakre, Vrushali Athawale, Tejaswini Fating

**Affiliations:** 1 Community Health Physiotherapy, Ravi Nair Physiotherapy College, Datta Meghe Institute of Higher Education and Research, Wardha, IND

**Keywords:** case report, chest wall, chemotherapy, treatment, ewings sarcoma, physiotherapy

## Abstract

Ewing sarcoma (ES) is a highly fatal bone and soft tissue cancer that predominantly impacts adolescents and children. Primary ES can occasionally manifest as a tumour on the chest wall. Treatment typically consists of radiation, local surgery, and polychemotherapy, all of which have acute and chronic side effects that can detrimentally affect survivors' quality of life (QOL). In this case study, we discussed the case of a 19-year-old female who came with chief complaints of chest pain, swelling on the right side of her neck, difficulty breathing, and pain in her right shoulder radiating to her right arm and forearm for one year. She was diagnosed with ES of the chest wall and underwent chemotherapy treatment for the same at our tertiary care hospital. Our aim was to find out the role of physiotherapy, considering the radiological, pathological, and clinical aspects of the disease while the patient is undergoing chemotherapy sessions, which has been highlighted and found to be effective in increasing satisfaction levels, adherence to the treatment, improving muscle strength, lung function, and overall QOL.

## Introduction

Ewing sarcoma (ES) and Ewing-like sarcomas are highly aggressive forms of round-cell tumours originating in the mesenchymal tissues, predominantly impacting paediatric and young populations [1]. It contributes to around 2% of childhood malignancies, standing as the second most frequent bone cancer in children following osteosarcoma, with its highest occurrence observed around the age of 15; this cancerous condition can manifest in any part of the body [2,3]. ES originates from soft tissue and bone, with the pelvis and lower extremities being the most common osseous. Metastases are observed in up to 25% of individuals at diagnosis, most often in the bone/bone marrow (40-45%) and lung (70-80%) [4]. They are identified genetically with the t(11;22) (q24:q12) chromosomal translocations (85% of cases). These tumours are characterized histologically by the presence of small, round cells that express high amounts of cluster of differentiation 99 (CD99) [5]. ES has essentially generic clinical manifestations. Patients may complain of confined pain, which may be followed by swelling and be misinterpreted as a small injury. The pain is usually mild, rising at night or after activity, although some individuals experience no pain at all. The accidental palpation of a firm lump may be the only symptom in the absence of pain. Nonetheless, pathological fractures are recorded in 10 to 15% of patients, and in advanced illness, nonspecific constitutive symptoms such as night sweats, fever, lethargy, and weight loss may occur [6]. It often spreads quickly. Within a few weeks, skeletal lesions tend to transform into massive tumours that occur in soft tissues [7].

It typically appears in the second decade of life, although instances have been reported from infancy to the eighth decade, with tumours identified in nearly every region of the body [8]. Because bone tumours are so aggressive, early detection is important. However, diagnostic delays are typical. Owing to late patient presentation, clinicians' low suspicion, and vague symptoms resembling common musculoskeletal injuries, it is difficult to make a reliable diagnosis in a timely manner. Plain radiography is the preferred diagnostic test [9]. An accurately done biopsy begins with the procedure of ensuring a bone cancer diagnosis, determining tumour grade, and guiding treatment. Currently, magnetic resonance imaging (MRI) is the foremost diagnostic tool for bone pain, particularly in children [10]. If the MRI results are unclear, projection radiography or computed tomography (CT) must be performed [11]. Multimodal therapeutic techniques, including radiotherapy, surgery, and intense multiagent chemotherapy, have led to a notable improvement in the survival rates of individuals diagnosed with localized illnesses. Tumor location, tumour age, volume, response to treatment, and metastatic disease sites were found to have significant predictive significance in various studies [12]. Current ES chemotherapy methods involve different combinations of the six medicines cyclophosphamide (CPM), doxorubicin (DOX), vincristine (VCR), etoposide (ETO), ifosfamide (IFO), and actinomycin-D (ACT-D). Local ES lesions are often managed with surgical excision, radiation, or both [13].

A rib is involved in around 10% of all ES cases. Incorporation of radiation therapy can cause lung and visceral damage, as well as exacerbate restrictive pulmonary illness. Traditional treatment options included the surgical removal of sections of the chest wall involving three or more ribs, coupled with radiation therapy. These types of therapy have resulted in problems such as decreased thoracic expansion, decreased respiratory function (forced expiratory volume in one second [FEV1] 80%), scoliosis, local deformity, and decreased quality of life (QOL) [14]. Cancer therapy can lead to a decline in physical fitness, decreased muscular strength, weariness, and a worse quality of life. It is critical to use physiotherapeutic interventions to alleviate the secondary problems associated with chest wall Ewing sarcoma (CWES) and chemotherapy.

Limited research exists concerning physiotherapy in these patients. As a result, our focus has been on incorporating concurrent physiotherapy during chemotherapy to improve the adherence of the patient to the treatment, increase satisfaction levels, prevent chest-related problems, improve muscle strength, and enhance overall QOL.

## Case presentation

Patient information

We outline the case of a 19-year-old female who was brought to our tertiary care hospital to seek management for her condition. The patient was all right a year before, when she developed chest pain, swelling on the right side of her neck, difficulty breathing, and pain in her right shoulder radiating to her right arm and forearm, which was insidious in onset and gradually progressive in nature. The patient also complained of tingling and numbness sensations over the right side arm and forearm, for which she was taken to a local hospital in Bhandara, where an X-ray was carried out and she was diagnosed with pleural effusion. She was managed conservatively at the same hospital and got no relief. Due to the persistence of symptoms, the patient came to our tertiary care hospital, where she had undergone certain investigations, like an X-ray, CT scan, and biopsy, and was diagnosed with Ewing sarcoma of the chest wall involving two ribs along with secondary pleural effusion, for which the patient underwent chemotherapy sessions (Table 1). She had no history of alcoholism. She had a history of decreased appetite and weight loss due to ongoing chemotherapy sessions. She neither gives a history of any comorbidities nor bladder or bowel complaints. During ongoing chemotherapy sessions, the patient complained of generalized weakness, chest pain, and breathlessness, for which physiotherapy rehabilitation was commenced with the proper tailor-made protocol for the patient.

**Table 1:** Chemotherapy drugs mg: milligram, gm: gram, IV: intravenous, D: day

Chemotherapy drugs	Dosage
Etoposide	100 mg/m^2^ (IV infusion) D1-D5
Ifosfamide	1.2 gm/m^2 ^(IV infusion) D1-D5
Mesna	200 mg/m^2^ (IV infusion) D1-D5

Diagnostic assessment

**Figure 1:**
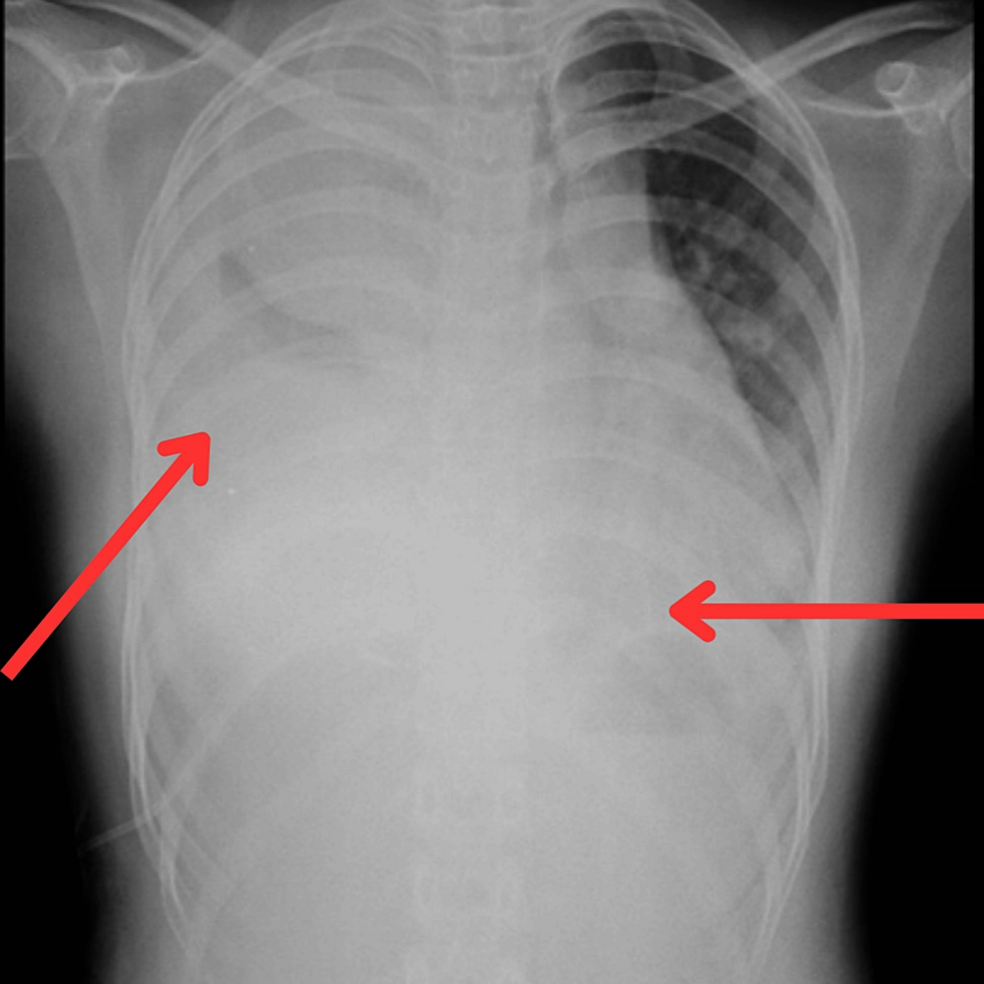
Chest X-ray (posteroanterior view) The arrow shows homogeneous opacity in the upper and lower zone of the right lung and the lower zone of the left lung along with the obliteration of both side costophrenic and cardiophrenic angles.

An X-ray, high-resolution computed tomography (HRCT) and biopsy examination were done. The X-ray revealed homogeneous opacity in the upper and lower zone of the right lung and the lower zone of the left lung along with the obliteration of both side costophrenic and cardiophrenic angles (Figure 1).

**Figure 2:**
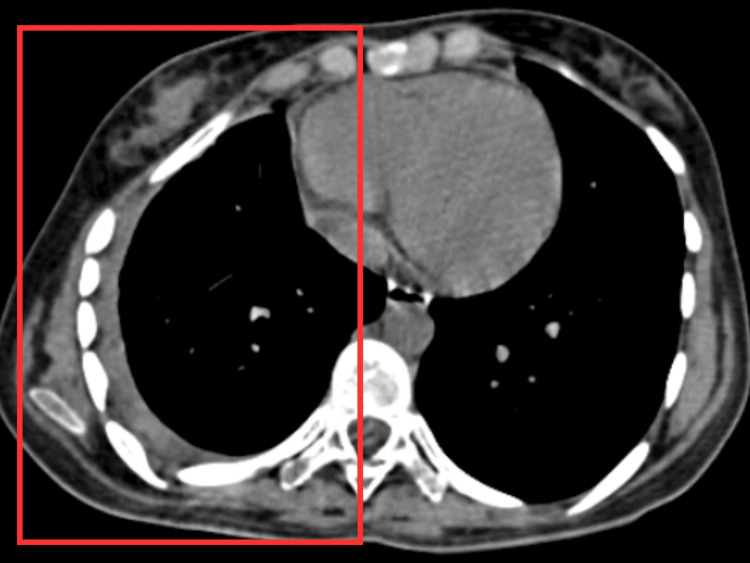
HRCT thorax HRCT: high-resolution computed tomography The square shows an ill-defined heterogeneously hyperdense lesion with hypodense areas and multiple calcific foci measuring approximately 7.4 × 5.8 × 5 cm [craniocaudal (CC) × transverse (TV) × anteroposterior (AP)] in right upper lobe adjacent to 1st and 2nd ribs, causing mild sclerosis of 1st rib

The high-resolution computed tomography (HRCT) thorax showed an ill-defined heterogeneously hyperdense lesion with hypodense areas and multiple calcific foci within measuring approximately 7.4 × 5.8 × 5 cm (craniocaudal {CC} × transverse {TV} × anteroposterior {AP}) in right upper lobe adjacent to 1st and 2nd ribs causing mild sclerosis of 1st rib (Figure 2).

**Figure 3:**
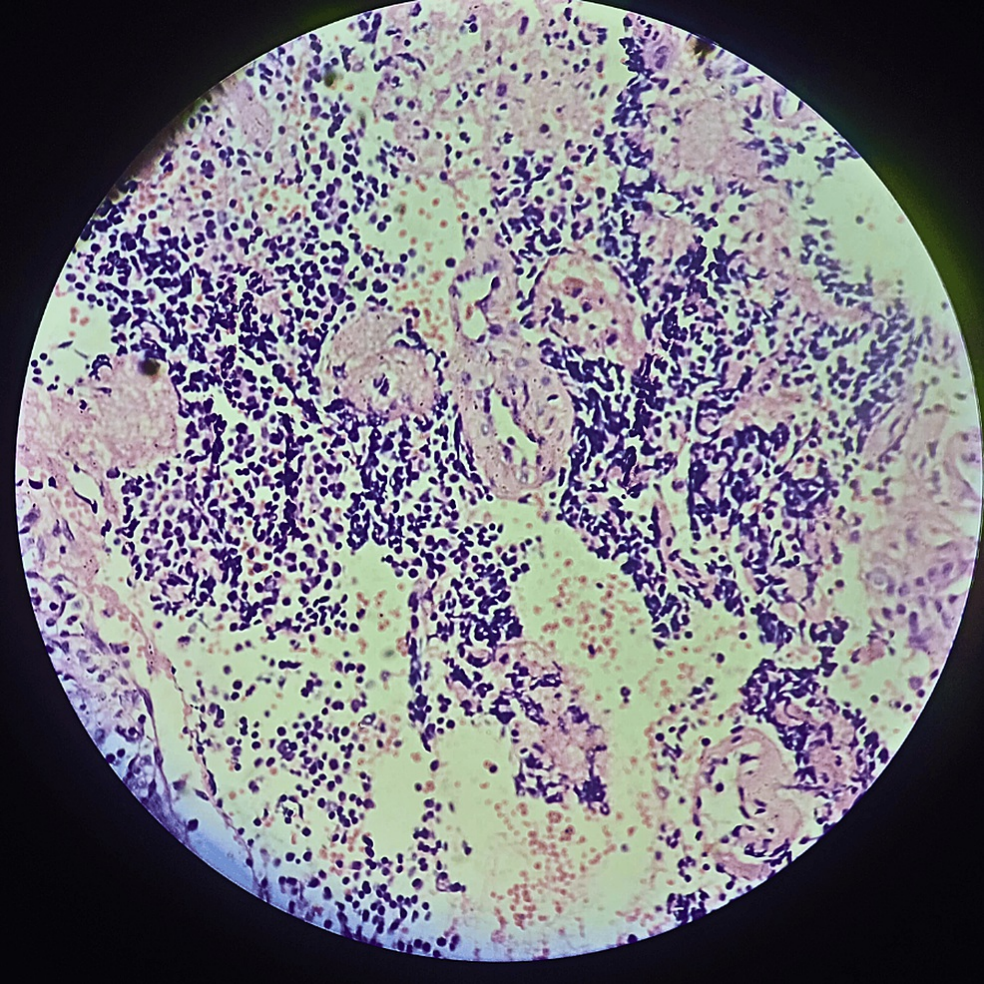
Histopathology report Biopsy revealed fragments of tumour tissue composed of small round-to-oval cells with round-to-oval hyperchromatic nuclei and scanty cytoplasm

The biopsy was performed as a diagnostic tool and the sample was taken from right-sided neck swelling (Figure 3). 

Physiotherapy assessment

Before the commencement of the examination, informed consent was obtained from the patient. She was cooperative, lethargic, and well-oriented to person, place, and time. On examination, the patient was afebrile and hemodynamically stable. The patient was seen in a sitting posture. She was ectomorphic in build, with a body mass index (BMI) of 15 kg/m^2^. On observation, swelling on the right side of her neck and bilateral supraclavicular retraction were noted. On examination, her chest expansion was found to be reduced at the nipple (1.5 cm) level. Pain was intermittent and dull in nature and the patient rated it as 4/10 on activity and 3/10 on rest according to the numerical pain rating scale (NPRS). The muscle strength (Table 2) was reduced in both the upper and lower limbs. The modified medical research council (mMRC) dyspnea grading was grade 2 [15]. On auscultation, air entry was reduced bilaterally. The patient was able to stand and walk independently.

**Table 2:** MMT of upper and lower limb MMT: Manual Muscle Testing 0: No contraction, 2: Full range of motion in gravity eliminated position, 3: Full range of motion against gravity position, -4: Initiates some but not complete range of motion against minimal resistance position, 4: Full range of motion against minimal resistance, 5: Full range of motion against maximal resistance

Limb	Muscles	Right	Left
Upper limb	Shoulder flexors	3/5	3/5
	Shoulder extensors	3/5	3/5
	Shoulder abductors	3/5	3/5
	Elbow flexors	3/5	3/5
Lower limb	Hip flexors	3/5	3/5
	Hip extensors	3/5	3/5
	Hip abductors	3/5	3/5
	Hip adductors	3/5	3/5
	Knee flexors	3/5	3/5
	Knee extensors	3/5	3/5
	Ankle dorsiflexors	3/5	3/5
	Ankle plantarflexors	3/5	3/5

Physiotherapy management

The physiotherapist devised particular exercises based on the oncologist's loading recommendations and the clinical condition of the patient (Table 3). The patient had no movement constraints throughout the chemotherapy infusion and was free to ambulate around the vicinity and participate in the rehabilitation session. The physiotherapy session was carried out during all inpatient chemotherapy sessions.

**Table 3:** Physiotherapy intervention NA: not applicable, reps: repetition, TENS: transcutaneous electrical nerve stimulation, cc: cubic centimetre

Problem identified	Goal	Intervention	Progression
Patient and family education	To enhance and maintain the patient’s positive attitude towards treatment for early recovery	The patient, along with his family was well-explained regarding his condition and was told about the importance of physiotherapy intervention	The home programme was explained
Breathlessness	To relieve breathlessness	Dyspnea-relieving position in side lying, sitting and standing	NA
Reduced chest expansion	To increase chest mobility	Thoracic expansion exercises (10 reps x 1 set, twice daily)	Segmental breathing exercise
		Spirometry (600 cc with 2 sec hold)	Spirometry (1200 cc with 2 sec hold)
Reduced ventilation	To improve the ventilation and oxygenation of the lung, to prevent fatigue	Diaphragmatic breathing exercise (10 reps x 1 set, twice a day)	15 reps x 1 set, twice a day
Early fatigue and anxiety	To reduce anxiety and promote relaxation	Pacing techniques, Jacobson relaxation technique (15 minutes twice a day	NA
Reduced muscle strength	To increase muscular strength of both upper and lower limb	Strengthening exercises for upper and lower limb (10 reps with 5 seconds hold x 1 set)	Strengthening exercises with 1/2 kg weight cuff (10 reps x 1 set)
Pain in the thoracic region	To relieve cancer-related pain	Low TENS (for 20 minutes)	NA
Reduce functional capacity	For increasing the functional capacity and fitness	Aerobic exercises, bedside walking (10 minutes)	30 minutes

**Figure 4:**
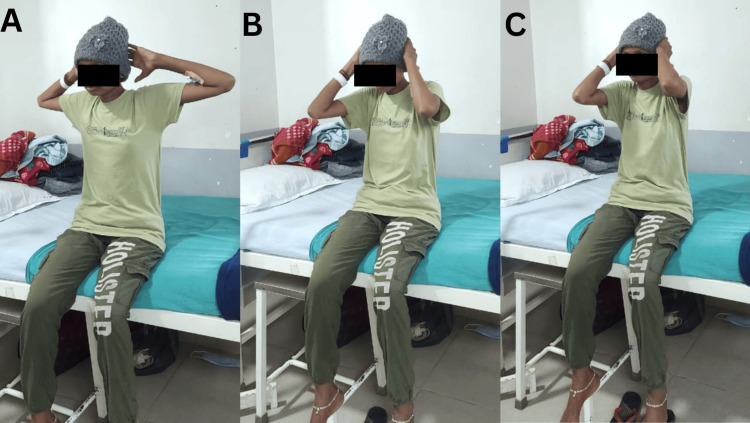
Patient performing a thoracic expansion exercise A: Initial position of thoracic expansion exercise B: Mid-position of thoracic expansion exercise C: End position of thoracic expansion exercise

Figure 4 shows the patient performing thoracic expansion exercises to improve chest mobility.

**Figure 5:**
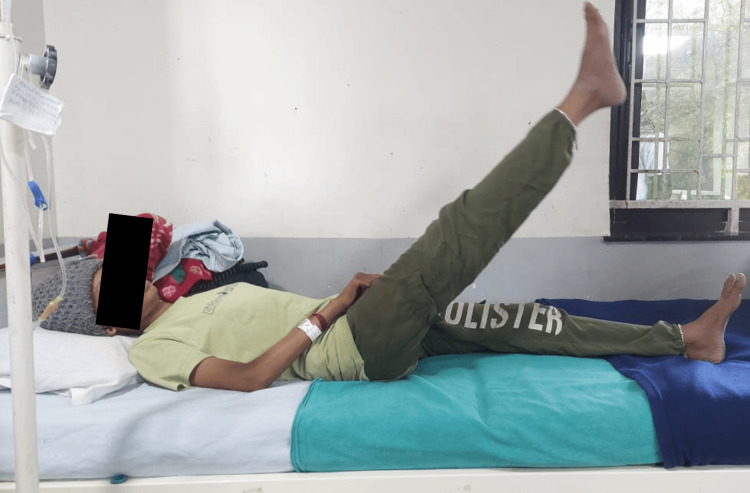
Patient performing lower limb strengthening exercise (SLR with a 5-second hold) SLR: Straight leg raise

Figure 5 shows the patient performing a lower limb strengthening exercise which included a straight leg raise (SLR) with a 5-second hold.

Outcome measures

The patient underwent an organized physical therapy protocol for four weeks. A follow-up was carried out after four weeks. The findings of the pre- and post-intervention manual muscle testing of the upper and lower limbs are shown in Table 4 and muscle strength was found to be improved.

**Table 4:** Pre- and post-intervention manual muscle testing of upper and lower limb 0: No contraction, 2: Full range of motion in gravity eliminated position, 3: Full range of motion against gravity position, -4: Initiates some but not complete range of motion against minimal resistance position, 4: Full range of motion against minimal resistance, 5: Full range of motion against maximal resistance

Limb	Muscles	Pre-intervention	Post-intervention
Upper limb	Shoulder flexors	3/5	-4/5
	Shoulder extensors	3/5	-4/5
	Shoulder abductors	3/5	-4/5
	Elbow flexors	3/5	-4/5
Lower limb	Hip flexors	3/5	-4/5
	Hip extensors	3/5	-4/5
	Hip abductors	3/5	-4/5
	Hip adductors	3/5	-4/5
	Knee flexors	3/5	-4/5
	Knee extensors	3/5	-4/5
	Ankle dorsiflexors	3/5	-4/5
	Ankle plantarflexors	3/5	-4/5

Table 5 shows pre- and post-intervention outcome measures scores which were found to be improved after following 4 weeks physiotherapy protocol.

**Table 5:** Outcome measures HAM-A: Hamilton anxiety rating scale, mMRC: modified medical research council, NPRS: numerical pain rating scale, SF12: 12-Item Short Form Survey, PCS: physical component score, MCS: mental component score The normal value of chest excursion at nipple level is 2–3 cm mMRC-: Grade 1: I get short of breath when hurrying on level ground or walking up a slight hill, Grade 2: On level ground, I walk slower than people of the same age because of breathlessness, or have to stop for breath when walking at my own pace [15]

Outcome measures	Pre-intervention	Post-intervention
Hamilton Anxiety Rating Scale (HAM-A)	24	14
Fatigue Severity Scale	48	28
mMRC grading	Grade 2	Grade 1
NPRS	On activity- 4/10	On activity- 2/10
	On rest-3/10	On rest-1/10
Chest excursion (at nipple level)	1.5 cm	2.5 cm
SF12	PCS-12: 21.50289	PCS-12: 41.10940
	MCS-12: 26.15473	MCS-12: 50.11787

## Discussion

In the above case, we saw the rehabilitation of a CWES patient who had secondary complications associated with chemotherapy such as generalized weakness, chest pain, breathlessness, and reduced satisfaction levels and adherence to the treatment. Our patient-tailored physiotherapy protocol showed immense progress after four weeks of treatment. For those with CWES, there are no official recommendations for physiotherapy programmes. There are a few research studies on the positive impact of physical rehabilitation on a patient's functioning that were carried out by an interdisciplinary team with other healthcare professionals. In this study, we have given strengthening exercises for both upper and lower limb weakness and found improved strength [16]. Ormel et al. stated that there is growing evidence that physical activity, in addition to cancer therapy, is both feasible and safe [17]. Exercise therapies have been shown to encourage individuals with a variety of cancers to have better lifestyles. Exercise therapies can often reduce common side effects associated with cancer therapy, such as fatigue related to the disease, improve QOL, and increase physical fitness in patients [17]. We also incorporated thoracic expansion exercises, spirometry, and diaphragmatic breathing exercises, which improved chest expansion, ventilation, and reduced breathlessness. Kaple et al. have also given pulmonary rehabilitation and found it to be effective in reducing chest complications and improving lung function [18].

Our study also focused on improving the adherence of the patient to the treatment, incorporating pacing techniques that reduced fatigue levels, and using aerobic exercises to improve functional capacity, which led to increased adherence of the patient to the physiotherapy. According to Erickson et al., cancer patients with bone tumours well tolerated concurrent intensive rehabilitation treatment during chemotherapy [19]. Findings revealed that nearly all cancer patients suffering from bone tumours were able to attend all the scheduled physiotherapy sessions, with a compliance rate of 100%, which was found to be greater than earlier studies on diverse oncological populations [19]. Because ES of the chest wall is rare, only a few case series of patients have been documented. Morri et al. stated that intensive physiotherapy, beginning with chemotherapy administration, is an effective therapeutic option for bone cancer patients who have demonstrated their ability to adhere to it positively [20]. The Hamilton Anxiety Rating Scale (HAM-A) and 12-Item Short Form Survey (SF12) were used as outcome measures, which showed improvement in the quality of life and satisfaction levels of the patient. Rehabilitation therapies should thus be encouraged within chemotherapy wards in order to enhance patients' levels of satisfaction and aid their functional recovery [20].

The current study has the limitation of not being generalizable and a lack of use of reliable instruments such as dynamometry for the evaluation of muscle strength. Further research is required to examine these findings completely. Chemotherapy toxicity, for example, might be studied further, and other prognostic variables unique to bone tumour patients could be discovered so that more specific rehabilitation programmes can be established.

## Conclusions

In this case study, we saw a patient with chest wall Ewing sarcoma who had secondary complications associated with chemotherapy such as generalized weakness, chest pain, breathlessness and reduced satisfaction levels and adherence to the treatment. The patient has shown good adherence to treatment, improved muscular strength, reduced chest complications and anxiety, and enhanced overall quality of life. Commencing vigorous physiotherapy simultaneously with chemotherapy is a feasible choice for patients with chest wall Ewing sarcoma. Encouraging rehabilitation therapies within chemotherapy units becomes vital in assisting patients towards functional recovery and increased satisfaction levels. 
